# Narrow thermal range restricts fertilization and early growth in the habitat‐forming seaweed *Durvillaea potatorum* (Phaeophyceae)—Implications for aquaculture and climate resilience

**DOI:** 10.1111/jpy.70147

**Published:** 2026-03-03

**Authors:** Vincent H. S. Yap, Wouter Visch, Catriona L. Hurd, Jeffrey T. Wright

**Affiliations:** ^1^ Institute for Marine and Antarctic Studies University of Tasmania Hobart Tasmania Australia; ^2^ Blue Economy Cooperative Research Centre Launceston Tasmania Australia

**Keywords:** climate change, Fucales, hatchery methods, irradiance, optimum temperature, thermal performance

## Abstract

The southern bull kelp *Durvillaea potatorum* is a key habitat‐forming macroalga in southeastern Australia and has been identified as a species of interest for sustainable seaweed aquaculture. However, the species is threatened by rising ocean temperatures and other anthropogenic factors. Assessing the thermal limits across different life stages of *D. potatorum* is therefore crucial for understanding its response to warming and optimizing future aquaculture practices. Using a full two‐factorial design, we tested the effects of a wide range of temperatures (~3.5–30.0°C) and two light regimes (zero/low light: 0/~40, and high light: ~120 μmol photons · m^−2^ · s^−1^) on the reproductive and early life stages of *D. potatorum* from Eaglehawk Neck, Tasmania. Gamete release, fertilization, and early growth after 15 min, 24 h, and 7 days, respectively, were assessed. Thermal performance curves revealed that fertilization was the most thermally sensitive stage, exhibiting the lowest thermal optimum (*T*_opt_ = ~12.85°C), and narrowest thermal breadth (*T*_br_ = ~2.5°C) independent of light conditions. Temperature and light had little to no effects on egg and sperm release, whereas early germling growth exhibited thermal optima of 16.33 and 14.50°C under low and high light treatments, respectively. These results indicate that fertilization conditions need to be closely monitored during the hatchery phase of aquaculture. In addition, natural *D. potatorum* populations may become increasingly susceptible to ocean warming due to impaired fertilization, potentially leading to demographic shifts and range contractions toward cooler waters.

**Abbreviations**
AIC: Akaike information criterion
OSTIA: Operational Sea Surface Temperature and Ice Analysis
RO: reverse osmosis
TPC: thermal performance curve

## INTRODUCTION

The impact of warming on macroalgal forests has been well described, with declines observed in 38% of the ecoregions examined (Krumhansl et al., 2016) and a greater likelihood of local extinctions and range contractions in low‐latitude trailing range‐edge populations (Smale, 2020). For example, there has been a 95% decline in the Laminarian kelp *Macrocystis pyrifera* around Tasmania, Australia, with intact forests now only in a handful of places in southern Tasmania (Butler et al., 2020; Johnson et al., 2011). Similarly, on the west coast of Australia, the fucoid *Scytothalia dorycarpa* contracted approximately 100 km south during a marine heatwave (Smale & Wernberg, 2013). Given that kelp and fucoids are canopy‐forming foundation species that support extensive biodiversity and productivity on temperate rocky reefs (Filbee‐Dexter & Wernberg, 2018; Steneck et al., 2003; Wernberg et al., 2011), understanding the mechanisms driving their thermal responses is critical to the preservation of marine ecosystems that are currently experiencing warming, including Australian waters. This is particularly the case for fucoid seaweeds, which are six times more speciose than the order Laminariales in the southern hemisphere, occupy more than half of Australia's coastline, yet are poorly represented in the literature (~20%; Bennett et al., 2015; Coleman & Wernberg, 2017).

Thermal performance curves (TPCs) are valuable tools for understanding the thermal tolerance and responses to climate change of a species (Kellermann et al., 2019). Quantifying biological rates with TPCs yields valuable parameter estimates including optimum, maximum, and minimum temperatures, as well as thermal breadths and safety margins (Padfield et al., 2021). For instance, TPCs revealed a narrow optimal thermal range for growth and sexual development of gametophytes in the Laminarian kelp *Lessonia corrugata* (Paine et al., 2021), whereas *Ecklonia radiata* gametophytes and sporophytes exhibited varying thermal optima for growth (Britton et al., 2024; Schwoerbel et al., 2024; Veenhof et al., 2024). Previous work on fucoids similarly documented significant effects of temperature on egg release, fertilization, and germling production and survival in *Sargassum* spp. (Chu et al., 2012; Kerrison & Le, 2016; Liu et al., 2013; Yeh et al., 2020, 2021; Zhao et al., 2008); fertilization and germling settlement and mortality in *Scytothalia dorycarpa* (Andrews et al., 2014); embryo growth and ontogenic development in *Hormosira banksii* (Clark et al., 2013, 2020; Miller et al., 2020); and germling growth and survival in *Fucus* spp. (Altamirano et al., 2003; Steen & Rueness, 2004). These works demonstrated strong effects of temperature on different early life stages of both Laminarian kelps and fucoids, highlighting the need to examine life stage‐specific thermal responses.

The southern bull kelp genus *Durvillaea* is a Fucalean seaweed endemic to the southern hemisphere, occurring in the intertidal and subtidal zones of wave‐exposed, temperate, rocky reefs (Velásquez et al., 2020). There are eight formally recognized species, two of which are endemic to Australia—*D. potatorum* and *D. amatheiae* (Weber et al., 2017)—occurring on exposed coasts of southeastern Australia including Tasmania (Fraser et al., 2009; Velásquez et al., 2020). *Durvillaea* has a diplontic, direct life cycle with free‐living male and female sporophytes that undergo oogamous reproduction (Clayton et al., 1987; Maier & Clayton, 1989). In southeastern Australia, *Durvillaea* is reproductively active from winter through to early spring, releasing large quantities of eggs and sperm into the water column, where fertilization occurs immediately (Clayton et al., 1987; V.H.S. Yap, unpub. data). As foundation species and ecosystem engineers, *Durvillaea* provide shelter, nutrition, and habitat complexity, and they control local diversity and ecological functioning for associated communities (Schiel, 2019; Taylor & Schiel, 2005; Thomsen et al., 2021; Thomsen & South, 2019). *Durvillaea potatorum* has also long been used by Indigenous Australians for a myriad of purposes, including cooking, medicine, clothing, shelter, ceremonial activities, and other domestic uses, indicating its cultural and historical significance (Hurd et al., 2023; Thurstan et al., 2018).

As well as being ecologically and culturally important, *Durvillaea* is of commercial interest in Australia (Kelly, 2020) due to its high alginate content (Kelly & Brown, 2000) and plant bio‐stimulant properties (Arioli et al., 2024; Mattner et al., 2018). However, there is currently no *Durvillaea* aquaculture industry—although nursery methods are being developed (E. Barry, unpub. data; Visch et al., 2026)—and the industry relies on the collection of beach‐cast *Durvillaea* to meet its demands. Anecdotal evidence suggests that the extents of both standing and beach‐cast *Durvillaea* in Tasmania have decreased in the last decade (Tasmanian Planning Commission, 2024; D. Delaney, pers. comm.). Long‐term monitoring of natural populations of Australian *Durvillaea* has shown significant declines of around 70% over 31 years and 30% over the last decade, likely due to both anthropogenic and climate‐driven factors (Johnson et al., 2025). Similarly, *Durvillaea* species in New Zealand (*D. antarctica*, *D. poha*, and *D. willana*) are highly susceptible to marine heatwaves and have become locally extinct in a small region in the northeast of the South Island (Thomsen et al., 2021). Notably, populations of *Durvillaea* appear to be in decline in the northern range edge of their distribution, with elevated temperatures seemingly an important factor (Nimbs et al., 2025). With warming rates two to four times the global average, southeastern Australia—including Tasmania—is considered a global‐warming hotspot projected to experience further warming and more frequent heatwaves (Hobday & Pecl, 2014; Malan et al., 2021; Oliver et al., 2018). The thermal tolerance of reproductive and early life stages of *Durvillaea* spp., however, has not been assessed. As beneficial as it is for potential future aquaculture applications, identifying these temperature limits is also crucial for understanding the persistence of natural *Durvillaea* populations in warming waters.

Other key factors influencing early life stages of fucoids are light and photoperiod (daylength). For example, egg release in *Fucus distichus* and *Silvetia compressa* as well as sperm release in *Sargassum ilicifolium* have been reported to be governed by light quality and quantity (Pearson et al., 2004; Pearson & Brawley, 1996, 1998; Yeh et al., 2020). Increased irradiance from approximately 10–20 to 90–100 μmol photons · m^−2^ · s^−1^ also enhanced growth of *Sargassum* spp. germlings (Yeh et al., 2021; Zhao et al., 2008). The aim of the current study was to assess the effects of temperature and light on gamete release, fertilization, and early germling growth of *Durvillaea potatorum* and to identify thermal optima for each life stage. We hypothesized that (1) gamete release, fertilization, and early germling growth will peak at temperatures that align with ambient seawater conditions during the species' primary reproductive period (late winter to early spring), and (2) different irradiance levels will enhance gamete release and early growth but not fertilization.

## MATERIALS AND METHODS

### Experimental design

#### Temperature

To manipulate temperature, a gradient table comprising a solid aluminum block (767 mm long × 540 mm wide × 40 mm thick) with 108 holes (diameter: 46 mm) arranged in a 9 × 12 grid was used (see Schwoerbel et al., 2024). The table allowed for 12 temperatures ranging among approximately 3.5 and 30.0°C (with slight variations in each experiment; see Table S1). The cool end of the temperature‐gradient table was maintained via an ethylene glycol/water loop (Valvoline Heavy Duty Coolant; CAS 107‐21‐1) regulated by a Ratek cooling coil and Ratek TH8000 heater set to 0.8, 0.3, and 0.5°C (for gamete release, fertilization, and early germling growth experiments, respectively). The warm end was maintained using a hot water bath regulated by a Ratek TH8000 heater set to 42, 46, and 35°C, respectively. The whole setup was located in a temperature‐controlled room set to 13 ± 1°C. Despite the minor differences in the temperature of the water bath, the temperatures of the gradient table were all within the intended range, with 12 different levels achieved for all experiments (Table S1).

#### Light

To manipulate light, four LED arrays providing a total irradiance of ~120 μmol photons · m^−2^ · s^−1^ were positioned 1 m below the temperature‐gradient table. For the gamete release and fertilization experiments, treatment levels were light (~120 μmol photons · m^−2^ · s^−1^) versus dark (gradient table holes were blocked using aluminum foil). For the early germling growth experiment, treatment levels were high (~120 μmol photons · m^−2^ · s^−1^) versus low (~40 μmol photons · m^−2^ · s^−1^) light, with the latter treatment created by layering black plastic mesh (mesh size = ~1.5 mm) over the holes. The light levels were selected based on the maximum photosynthetically active radiation absorbed by blade tissues of field‐collected *Durvillaea potatorum* juveniles (~120 μmol photons · m^−2^ · s^−1^; M. Pelletier, unpub. data). Further, gametophyte growth in subtidal kelps in Tasmania—*Lessonia corrugata* (Paine et al., 2021) and *Ecklonia radiata* (Schwoerbel et al., 2024)—was highest between 40 and 120 μmol photons · m^−2^ · s^−1^. Thus, our light treatments represented realistic high and low levels for the growth of *Durvillaea* germlings in this region. A 12:12 light:dark photoperiod was used for all experiments.

To obtain a fully crossed design with 12 temperature levels and two light levels, four or five replicates were used per treatment combination (as in Paine et al., 2021). However, several replicates were removed from the analyses due to handling errors, which resulted in three replicates for the following treatments: the 5.9°C‐Light, 10.3°C‐Light, and 26.3°C‐Dark treatments for the egg release experiment; the 8.0°C‐Dark, 14.3°C‐Light, 22.0°C‐Light, and 23.8°C‐Dark treatments for the sperm release experiment; and the 14.3°C‐Light treatment for the fertilization experiment.

### Sampling and experimental procedures

Reproductive *Durvillaea* thalli were collected via snorkeling from Clydes Island, Eaglehawk Neck (43°00′35″ S, 147°56′29″ E). Average monthly sea surface temperatures for the site were obtained from the Operational Sea Surface Temperature and Ice Analysis (OSTIA) database between 1 January and 31 December 2024 (Copernicus Marine Service, 2025). Blade tissues were collected from the distal end of male and female adult *Durvillaea* from the intertidal zone. Samples collected from this depth would have primarily been *D. potatorum* (Velásquez et al., 2020).

For all three experiments, each experimental unit consisted of a 70‐mL plastic jar filled with filtered, autoclaved seawater. Jars were placed in the gradient table ahead of time so that they were at the desired temperature when the experiment began, although this was not done for the early growth experiment (see below).

#### Effects of temperature and light on gamete release

On July 24, 2024, a total of 192 blade tissue discs (~2.5 cm^2^ each) were collected in situ from 96 different male and female individuals using a metal hole punch. These were placed immediately into individual plastic containers and transferred back to the lab on ice. Upon arrival, blade tissue discs collected from females were washed with high purity, reverse osmosis (RO) water to remove contaminants and any eggs that might have been released prior to assessment and had adhered to the blade surface. The discs were promptly placed into individual jars—filled with 20 mL of seawater—in the gradient table. After 15 min, blade discs were removed from each jar, and 0.5 mL of absolute ethanol was added (final concentration of ~10%) to preserve samples for counting later. These steps were repeated for tissue discs collected from males using new jars.

For females, eggs were counted in a Sedgwick‐Rafter counting chamber. Specifically, 1 mL of egg suspension was added to the chamber, and eggs per sample were quantified by counting 20 or 40 of the 1‐mm^2^ squares of the counting chamber (1 mm depth, i.e., 20 or 40 mm^3^ total) depending on egg density. This was performed in triplicate for each replicate and the average number of eggs released per cm^2^ blade tissue determined, taking into account both sides of the discs since blades have conceptacles on both sides.

For males, spermatozoids were quantified in a hemocytometer by adding 250–300 μL of sperm suspension and counting them in an area of 0.02 mm^3^. Similarly, this was performed in triplicate, averaged, and the number of spermatozoids released per cm^2^ blade tissue determined.

#### Effects of temperature and light on fertilization

On 22 August 2024, individual blade tissues (~100 cm^2^ each) were collected from 10 different male and female individuals. Once collected, these tissues were placed on ice in the dark overnight. The following day, the tissues were washed with RO water and gametes were released. This was done separately for males and females; eggs were released first due to the much shorter viability of sperm. To release eggs, the 10 female blades were placed in a plastic tray with 800 mL of ~14°C filtered, autoclaved seawater and placed under light in a laminar‐flow cabinet. After 15 min, the blades were removed, and the egg suspension was decanted into a beaker. These steps were repeated for the 10 male blades. Immediately after obtaining the sperm suspension, 5 mL each of the egg and sperm suspension were transferred into individual jars in the gradient table. These jars were filled with 20 mL of seawater prior to the addition of gametes. A subsample from both gamete suspensions was obtained and counted using the same method as for the gamete release experiment to determine the sperm:egg ratio (~1300:1).

Fertilization was assessed 24 h post gamete mixing. Four different photos of the bottom of each jar were taken using a compound microscope (Nikon Eclipse Ci) and a camera (Nikon DS‐Ri2). The number of zygotes (fertilized eggs) and unfertilized eggs per photo was counted. The development of a protuberance at the rhizoid pole indicates successful karyogamy (Maier, 1997); fertilization rate for each replicate was thus specified as average percentage (pooled across four photos) of fertilized (rhizoid‐initiated) to unfertilized eggs after 24 h.

#### Effects of temperature and light on early growth

On August 28, 2024, individual blade tissues (~100 cm^2^ each) were collected from 10 different male and female individuals. Once collected, these were placed on ice and in the dark overnight. The following day, male and female tissues were washed separately with RO water and soaked in 10% Betadine solution for 2 min to eliminate contaminants/pathogens (and previously released gametes) on the blades.

Based on findings from the fertilization experiment, modifications were made to enhance fertilization success. Firstly, the sperm:egg ratio was altered by adjusting the volume of the gamete suspensions used. (The ratio for this experiment was approximately 450:1.) Female blades were placed in a plastic tray with 600 mL of ~12.5°C filtered, autoclaved seawater to release eggs under light in a laminar‐flow cabinet, followed by male blades to release sperm (in a separate tray). After 15 min, blades were removed from trays and egg and sperm suspensions decanted into separate beakers. Gametes were mixed by transferring 2.5 mL each of the egg and sperm suspension into individual jars previously filled with 20 mL of seawater. Secondly, to ensure standardized fertilization at the optimum temperature and light levels identified in the fertilization experiment, the jars were kept overnight in a dark, temperature‐controlled room set to 12.5 ± 1°C.

The next day, a subsample of replicates was assessed under a microscope (Nikon Eclipse Ci) to ensure fertilization had occurred (~80%–95%; see Figure S1b) before carefully decanting the seawater in each jar to remove excess, free‐floating gametes (fertilized eggs, i.e., zygotes, would have adhered to the bottom of the jar; Kevekordes & Clayton, 1999). The jars were then carefully rinsed with RO water before being replenished with 50 mL of ~12.5°C filtered, autoclaved seawater and placed in the gradient table. The replenished seawater was also inoculated with F10 nutrient media, a diluted but nutrient‐replete version of F2, which is the standard nutrient media used in seaweed cultivation.

After a week, photos of the bottom of each jar were taken using an inverted microscope (Nikon Eclipse Ts2) with a camera attached to it. For each replicate, the length of the longest 10 germlings was measured manually in ImageJ and growth estimated as the average increase in germling length after 7 days.

### Data analysis

A thermal performance curve was plotted for each light level for each experiment (gamete release, fertilization, and early germling growth) using the R packages rTPC and nls.multstart (Padfield et al., 2021). Nine models that included biologically relevant parameters, namely thermal optimum (*T*_opt_), were fit to each dataset. Briefly, each model followed a pattern typical of TPCs in which performance increased from low temperatures to an optimum/maximum rate (*R*_max_; at *T*_opt_) before declining rapidly with further temperature increase (Britton et al., 2024). The best model was chosen based on Akaike information criterion (AIC); however, models that displayed illogical/irrelevant patterns and parameter values despite low AIC were excluded (Table S2; Figure S2). Model uncertainty and confidence intervals for model parameters (i.e., *T*_opt_) were estimated using case resampling bootstrapping via the R package car. Thermal breadth (*T*_br_), which is defined as the temperature range where *R* ≥ 0.8 × *R*_max_, was also calculated using functions in the rTPC package (see figure 1 in Schwoerbel et al., 2024).

Differences in gamete release, fertilization, and early germling growth between the two light treatments/levels at the temperature closest to the model‐estimated *T*_opt_ were assessed using the non‐parametric Mann–Whitney *U* Test (Wilcoxon rank‐sum) given the small sample size, although data for all three experiments were screened for normality prior to testing (Shapiro–Wilk test; *W* = ~0.85–0.96, *p* > 0.05). All statistical tests were conducted in R v. 4.2.1 (R Core Team, 2022) with α = 0.05.

## RESULTS

**FIGURE 1:**
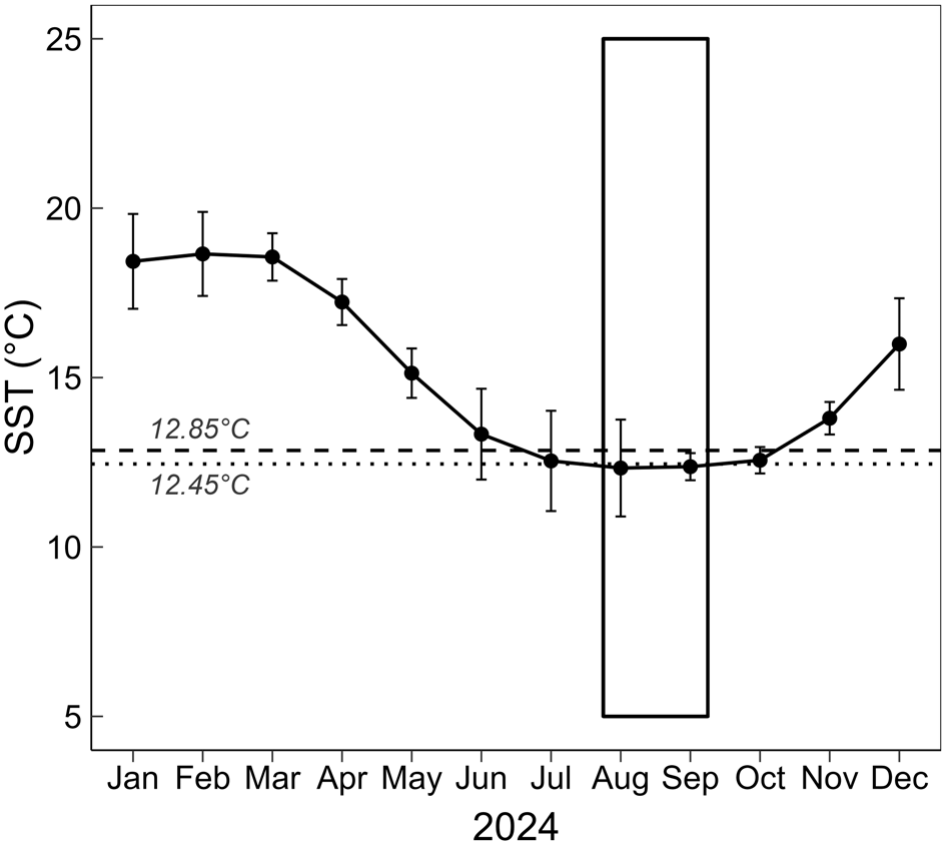
Monthly sea surface temperatures (mean ± *SD*) for Clydes Island, Eaglehawk Neck, Tasmania, Australia (43°00′34.7″ S, 147°56′29.0″ E) obtained from the Operational Sea Surface Temperature and Ice Analysis (OSTIA) database between January 1 and 31 December 31, 2024 (Copernicus Marine Service, 2025). The dashed and dotted lines represent the experimental thermal optimum for fertilization in *Durvillaea potatorum* (12.85°C) and the average temperature from July to October (12.45 ± 1°C; mean ± *SD*), respectively. The rectangle box indicates the period during which all three experiments were conducted.

Based on the 365‐day satellite data (OSTIA), average sea surface temperature at the sampling site (Clydes Island, Eaglehawk Neck) was the lowest, that is, ~12.35°C, at the time the three experiments were conducted (August–September). The highest mean temperatures at the site occurred from January to March (18.4–18.6°C; Figure 1).

### Effects of temperature and light on gamete release

**FIGURE 2:**
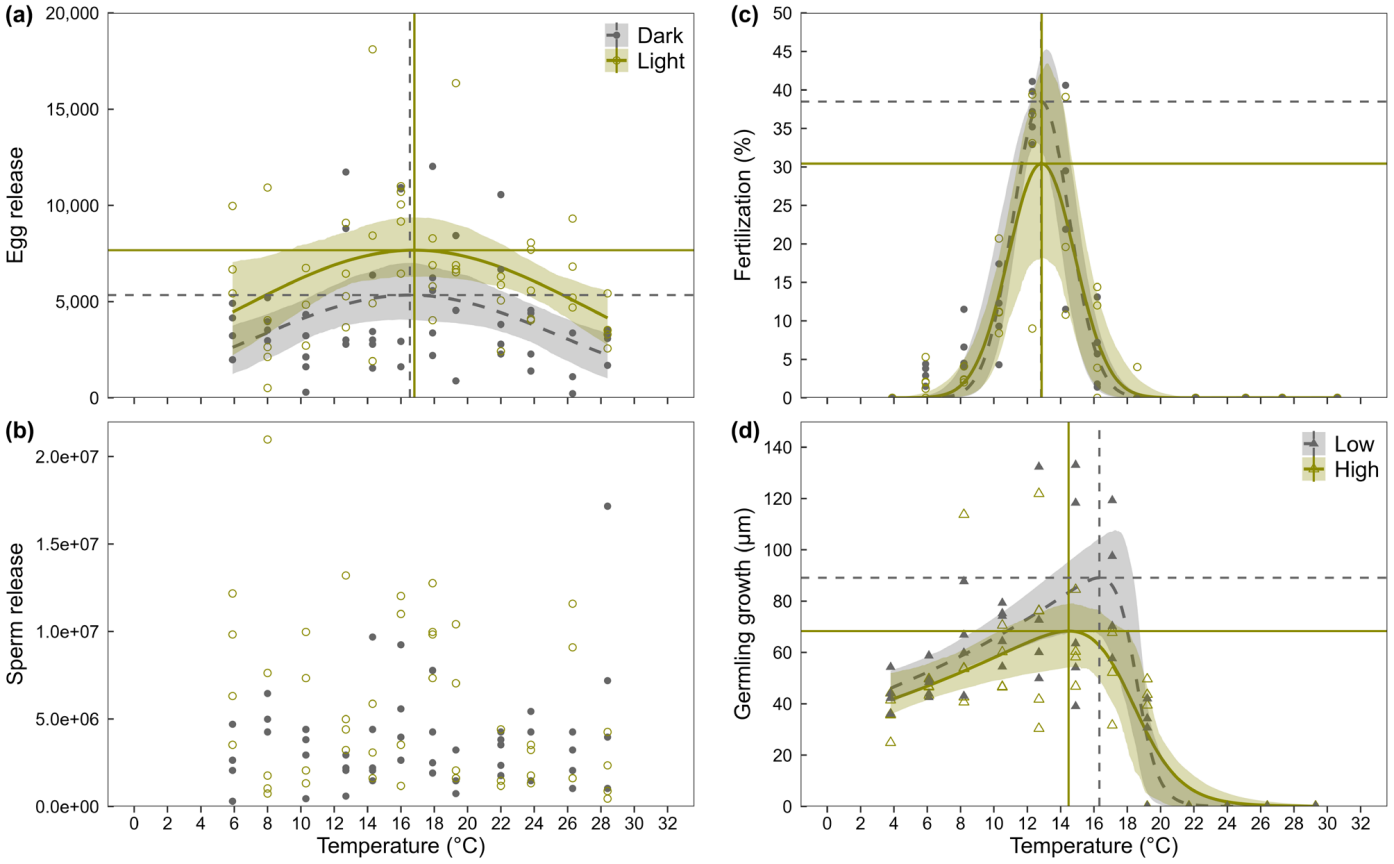
Thermal performance curves for (a, b) gamete release after 15 mins, (c) fertilization after 24 h, and (d) early growth of germlings after 7 days for *Durvillaea potatorum* under dark versus light treatment (a–c; 0 vs. ~120 μmol photons · m^−2^ · s^−1^; filled vs. unfilled circles), or low and high light (d; ~40 vs. ~120 μmol photons · m^−2^ · s^−1^; filled vs. unfilled triangles). Shaded areas correspond to the bootstrapped 95% confidence intervals of model fits. Vertical lines indicate model‐based estimates of thermal optima and horizontal lines indicate the corresponding maximum rates (dotted lines: Dark/low light, solid lines: Light/high light; Table 1). *n* = 3–5 for each temperature and light treatment combination.

Egg release was weakly affected by temperature for both dark and light treatments. This was reflected in the wide thermal breadths (*T*_br_ = 11.96 and 13.98°C, respectively) and slight declines at both low and high temperatures relative to the thermal optima (*T*_opt_ = 16.54 and 16.81°C, respectively; Table 1; Figure 2a). Maximum egg release after 15 min was marginally higher in the light treatment compared to the dark treatment with confidence intervals overlapping slightly (*R*_max_ = 7669 and 5338 eggs per cm^2^, respectively; Table 1; Figure 2a). The difference in egg release between the two light treatments closest to *T*_opt_ was not significant (Wilcoxon rank‐sum: *W* = 8, *p* = 0.7302).

**TABLE 1:** Model parameters fitted to thermal performance curves under dark and light treatments (egg release, sperm release and fertilization), or under low and high light (early germling growth).

Response	Light level	Model	*T*_opt_	*R*_max_	*T*_br_
Egg release	Dark	Gaussian	16.54 (13.85–18.93)	5338 (3850–7095)	11.96 (8.42–19.36)
	Light	Gaussian	16.81 (10.36–19.06)	7669 (6342–9533)	13.98 (9.99–22.50)
Sperm release	Dark	n/a	n/a	n/a	n/a
	Light	n/a	n/a	n/a	n/a
Fertilization (%)	Dark	Gaussian	12.83 (12.31–13.26)	38.48 (33.26–45.73)	2.27 (1.84–2.86)
	Light	Gaussian	12.86 (12.24–13.65)	30.43 (16.03–39.68)	2.67 (2.14–3.85)
Early germling growth (μm)	Low	Pawar	16.33 (14.05–17.56)	89.10 (69.05–105.76)	6.53 (5.05–11.98)
	High	Pawar	14.50 (12.02–17.29)	68.34 (55.23–79.38)	8.37 (6.55–15.14)

*Note*: Final models were selected based on Δ AIC scores (Table S2). *T*_opt_ = thermal optimum and *R*_max_ = maximum rate at *T*_opt_. Values are model estimates of each parameter with 95% confidence intervals in parentheses.

Conversely, sperm release appeared largely unaffected by temperature and light treatments (Figure 2b). None of the eight fitted TPC models (Pawar model was not able to fit the data) were adequate or visually representative (Figure S2b), and subsequently there were no parameter estimates (*T*_opt_, *T*_br_, *R*_max_) for sperm release (Table 1; Figure 2b).

### Effects of temperature and light on fertilization

Fertilization was strongly affected by temperature. Thermal optima were 12.83 and 12.86°C for dark and light treatments, respectively, with a steep decrease on either side of these optima, reflected in the narrow thermal breadths (*T*_br_ = 2.27 and 2.67°C, respectively; Table 1; Figure 2c). Above 16°C, there was virtually no fertilization observed (Figure 2c). The maximum fertilization rate after 24 h was slightly higher in the dark treatment compared to the light treatment (*R*_max_ = 38.48% and 30.43%, respectively), albeit with substantial overlap in confidence intervals (Table 1; Figure 2c). Driven by a single outlier in the light treatment, the difference in fertilization between the two light treatments closest to *T*_opt_ was not significant (Wilcoxon rank‐sum: *W* = 14, *p* = 0.4127).

### Effects of temperature and light on early germling growth

Early germling growth was also strongly affected by temperature, with relatively wide thermal breadths for both low and high light treatments (*T*_br_ = 6.53 and 8.37°C, respectively). The thermal optimum differed between the light levels (*T*_opt_ = 16.33 and 14.50°C, respectively), although confidence intervals mostly overlapped (Table 1; Figure 2d). No live germlings were observed beyond 20°C. Maximum germling growth after 7 days was higher under low light compared to under high light (*R*_max_ = 89.10 and 68.34 μm, respectively) with some overlap in confidence intervals (Table 1; Figure 2d). The difference in early germling growth between the two light levels closest to *T*_opt_ was not significant (Wilcoxon rank‐sum: *W* = 4, *p* = 0.3429).

## DISCUSSION

We identified fertilization as the most thermally sensitive reproductive stage in *Durvillaea potatorum*, with narrow thermal breadths, comparable thermal optima between dark and light treatments (12.83 and 12.86°C, respectively), and virtually no fertilization above 16°C. Early growth of *D. potatorum* germlings was also dependent on temperature but exhibited higher thermal optima and broader thermal breadths than fertilization (*T*_opt_ = 16.33 and 14.50°C under low and high light, respectively), with no germlings surviving at temperatures above 20°C. In contrast, gamete release was largely unaffected by temperature and light; egg and sperm release were consistently observed across the entire tested thermal range and under both light conditions, although there was weak evidence of a thermal optimum for egg release. Overall, our results indicate high thermal sensitivity in key early life‐cycle traits in *Durvillaea* (i.e., fertilization and early germling growth), highlighting implications for aquaculture and the species' response to ocean warming.

The estimated thermal optima for fertilization in *Durvillaea potatorum—*with or without light—correspond to the average ambient seawater temperatures recorded at the sampling site from July to October (Figure 1), which is when natural *D. potatorum* populations in Tasmania are most reproductive (V.H.S. Yap, unpub. data). Previously reported thermal optima for fertilization in other fucoids—for example, *Scytothalia dorycarpa* (Andrews et al., 2014), *Sargassum* spp. (Kerrison & Le, 2016), and *Fucus* spp. (Steen & Rueness, 2004)—were also within ambient seawater temperature ranges during their respective reproductive periods. Other species such as *Phyllospora comosa*, which reproduces year round, exhibited comparable germination rates across a relatively wider thermal range (15–21°C; Cumming et al., 2019). Evidently, taxa with more prolonged reproductive periods exhibit broader thermal windows for fertilization, and the winter‐reproducing *D*. *potatorum* (V.H.S. Yap, unpub. data) has exhibited one of the narrowest known thermal breadths for fertilization among fucoids, reflecting the limited cool water temperature range experienced by this genus (Nimbs et al., 2025).

The narrow thermal tolerance of *Durvillaea potatorum* fertilization may be attributable to reduced sperm viability at sub‐optimal temperatures. Spermatozoids can fertilize eggs only within the time period in which they are viable, and this period is governed by factors such as propagule size, motility, and velocity (Esposito et al., 2020; Li et al., 2013; Nardelli et al., 2025). Although some studies showed enhanced sperm motility of external fertilizers at higher water temperatures, experiments that incorporated a broad temperature range typically observed that sperm velocity peaked at intermediate temperatures, and any increase past the optima escalated metabolic rates and rapidly depleted finite energy resources (Wang & Gunderson, 2022). Furthermore, *D. potatorum* spermatozoids are smaller (~4 μm) than those of many other fucoids (e.g., ~10 μm in *Fucus* spp.), suggesting that they have less energy reserves and a reduced swimming capacity (Brawley et al., 1999; Hatchett et al., 2022; Kinoshita‐Terauchi et al., 2021). In fact, *D. potatorum* spermatozoids cease activity within a few minutes after release (Clayton & Ashburner, 1994; Doblin & Clayton, 1995). The limited energy supply and short sperm mobility period, coupled with impaired viability at unfavorable temperatures, likely contributed to the narrow thermal window for *D. potatorum* fertilization observed in this study.

Compared to fertilization, early germling growth of *Durvillaea potatorum* exhibited higher thermal optima (16.3 and 14.5°C for low‐ and high light treatments, respectively). Yet overall, these aligned with the thermal optima for early growth of laminarian kelps in Tasmania, that is, *Lessonia corrugata* gametophytes and juvenile sporophytes of *Ecklonia radiata* (15.7–17.9 and ~16°C, respectively; Britton et al., 2024; Paine et al., 2021). Although reproduction in these two species (i.e., sori production and zoospore release) occurs mostly year round, it generally also peaks in the cooler months (Hurd et al., 2023; Mabin et al., 2013; Nardelli et al., 2025; Tatsumi et al., 2022). An increase in temperature—to a certain extent—can enhance metabolic processes like photosynthesis and nutrient uptake, thereby increasing growth (Allakhverdiev et al., 2008; Clark et al., 2020). Beyond the thermal optimum, physiological processes such as denaturation of heat‐labile proteins and impairment of the photosynthetic and carbon‐fixation apparatuses take place, causing structural collapse and ultimately impeding survival and growth (Andrews et al., 2014; Clark et al., 2013; Cruces et al., 2013; Davison, 1991). For week‐old *D. potatorum* germlings, these processes are likely triggered at temperatures above approximately 14–16°C, at which growth declines drastically. Notably, this documented record of growth data for *D. potatorum* showed germlings growing up to a maximum length of approximately 89 μm in 1 week (i.e., ~13 μm · d^−1^; Table 1). This was much lower than germling growth rates reported for other fucoids, for example, ~49 μm · d^−1^ for *Hormosira banksii* (Clark et al., 2013), indicating that *Durvillaea*, although physically much larger, appears to be relatively slow growing, at least during its early life stages.

The limited effect of temperature on gamete release in *Durvillaea potatorum* aligned with results from previous literature. Specifically, a review comprising 71 temperate seaweed species reported that temperature was a good predictor of gamete release only in two species: the fucoids *Ascophyllum nodosum* and *Sargassum muticum* (de Bettignies et al., 2018). The effect of light on gamete release and fertilization of *D. potatorum* was also negligible. The limited impact of irradiance on egg release was similarly reported in *Sargassum* spp. (Liu et al., 2013; Yeh et al., 2020). The occurrence of *Durvillaea* on relatively wave‐exposed shores suggests that other exogenous factors, including water movement, lunar or tidal cycles, photoperiod, and desiccation (Brawley et al., 1999; Cumming et al., 2019; de Bettignies et al., 2018; Hatchett et al., 2022; Pearson & Serrão, 2006), may govern gamete release in the species. Additionally, fertilization occurs rapidly in *Durvillaea*, with spermatozoid nuclei, mitochondria, and flagella inside the egg membrane 2 min after gamete mixing (Clayton & Ashburner, 1994). Consequently, ambient light conditions are unlikely to influence the non‐phototactic *D. potatorum* spermatozoids (Clayton et al., 1987) between release and syngamy and thus have little to no effect on fertilization.

Early growth in *Durvillaea potatorum* appeared to be the life stage most influenced by the light treatment in this study; germling growth and thermal optimum were both lower—albeit marginally—under high light compared to under low light. Our resulted aligned with those for *Ascophyllum nodosum* and *Sargassum* spp., for which optimal irradiance for growth of germlings during the first 2 to 4 weeks of development was reported to range between 40 and 60 μmol photons · m^−2^ · s^−1^, with 100 μmol photons · m^−2^ · s^−1^ identified as the lethal threshold (Chai et al., 2014; Coutinho et al., 2025; Hales & Fletcher, 1989; Yan & Zhang, 2014). In fact, high germling production has occurred at low light and high temperature (*Sargassum muticum*; Kerrison & Le, 2016). Compared to adults, juvenile seaweeds can typically tolerate less light (Ruiz Martínez et al., 2024), and microscopic germlings of *D. potatorum—*like those of *S. muticum* (Kerrison & Le, 2016)—appeared sensitive to high light. The irradiance level used in our high light treatment (120 μmol photons · m^−2^ · s^−1^) very likely exceeded the photosynthetic needs of days‐old *Durvillaea* germlings, resulting in photoinhibition. Under low light conditions, energetic costs of photoinhibition decreases, freeing up resources for other metabolic processes, including photosynthesis, which may have enhanced the growth of *D. potatorum* germlings, even at elevated temperatures. Better germling growth in *D. potatorum* in low light is expected given its reproductive period (i.e., winter) is characterized by relatively low light availability, and *D. potatorum* occurs in low light environments beneath dense canopies of large, thick‐bladed adults (Velásquez et al., 2020).

### Implications for cultivation and climate resilience

Being the most thermally sensitive stage, the fertilization of *Durvillaea potatorum* should be closely regulated during the hatchery phase. Specifically, both gamete release and fertilization should be conducted in seawater maintained at the thermal optimum for fertilization—12.8°C—whereas light control is unnecessary at these stages. To facilitate optimal early germling growth, we propose increasing the temperature to 15–16°C at 1 day post fertilization. Although *D. potatorum* germlings seemed to grow better in low light conditions (≤40 μmol photons · m^−2^ · s^−1^) within the first week, the optimal irradiance for growth of older germlings could be higher (Coutinho et al., 2025; Yan & Zhang, 2014). For instance, Yeh et al. (2021) observed that high irradiance (100 vs. 20 and 50 μmol photons · m^−2^ · s^−1^) significantly improved the growth of *Sargassum ilicifolium* germlings in weeks 4–8 but not in week 1. Experiments with longer timeframes are necessary to confirm this and to better understand the light (and temperature) requirements for older *D. potatorum* germlings.

The narrow thermal range for fertilization in *Durvillaea potatorum* shown in this study indicates it is vulnerable to ocean warming. Sea surface temperatures are projected to increase on average by 0.86–2.89°C over the next 50 years (Fox‐Kemper et al., 2023), pushing the fertilization success of *D. potatorum* to the upper edge of its range, which will likely have implications for recruitment and long‐term persistence. Although gamete release and growth can occur at temperatures higher than the thermal optimum for fertilization, a prolonged overlap between ambient ocean temperatures and the lethal limits of a single ontogenetic stage—fertilization in this case—is enough to modify a population's demographic structure and result in range contractions toward cooler environments (Andrews et al., 2014). This result was echoed by a recent genomic study by Nimbs et al. (2025) who examined the thermal adaptive capacity of the sister species *D. amatheiae* in southeastern mainland Australia and observed little remaining adaptive capacity, especially among the warmest populations, implying a susceptibility to range contractions amid the ongoing climate crisis. Nonetheless, the thermal window for fertilization in warm‐edge populations of *D. potatorum* is unknown; they may possess higher thermal optima for fertilization due to local adaptation, particularly given the higher water temperatures experienced during peak reproductive periods. Several Australian seaweed species distributed along a latitudinal thermal gradient, including *Hormosira banksii* (Miller et al., 2020) and *Ecklonia radiata* (Britton et al., 2024; Veenhof et al., 2024) showed growth and developmental responses consistent with local adaptation to warmer waters. Notably, there is a potential for translocation of warm‐adapted *D. potatorum* populations to cooler waters for both cultivation and conservation purposes, but further research is needed to test these hypotheses. We recommend that future studies assess early life development—namely fertilization—in *Durvillaea* populations inhabiting warmer coastlines, particularly along mainland Australia.

## AUTHOR CONTRIBUTIONS

**Vincent H. S. Yap:** Conceptualization (equal); data curation (lead); formal analysis (lead); investigation (lead); methodology (lead); visualization (lead); writing – original draft (lead); writing – review and editing (lead). **Wouter Visch:** Conceptualization (equal); investigation (supporting); methodology (supporting); resources (equal); supervision (supporting); writing – review and editing (equal). **Catriona L. Hurd:** Conceptualization (equal); methodology (supporting); resources (equal); supervision (supporting); writing – review and editing (equal). **Jeffrey T. Wright:** Conceptualization (equal); funding acquisition (lead); investigation (supporting); methodology (supporting); resources (equal); supervision (lead); writing – review and editing (equal).

## FUNDING INFORMATION

The authors acknowledge the financial support of the Blue Economy Cooperative Research Centre, established and supported under the Australian Government's Cooperative Research Centres Program, grant number CRCXX000001 (previously 20180101).

## Supporting information


**Figure S1.** Microscope images of one of the samples from the (a) fertilization experiment, and (b) early germling growth experiment, 24 h after fertilization, indicating ~35–40 and >95% fertilization success, respectively. Fertilization occurred at ~12.5°C in darkness for both samples.

**Figure S2.** All thermal performance curves models fit to (a) egg release, (b) sperm release, (c) fertilization, and (d) early germling growth of *Durvillaea potatorum* at each light level. Nine models were fit to each response except sperm release, which could not be fit with a Pawar model.

**Table S1.** The 12 temperature levels measured in the temperature‐gradient table prior to each experiment.

**Table S2.** Details of all thermal performance curve models fitted to egg release, fertilization, and early germling growth of *Durvillaea potatorum*. The model with the lowest ΔAIC (difference from model with lowest AIC) was preferentially selected. Despite their low ΔAIC values, some models (*) were excluded due to illogical patterns and/or parameter values (see Figure S2) and the model with next lowest Δ AIC was chosen instead. Sperm release was not modeled as none of the fitted thermal performance curve models were adequate or visually representative (see Figure S2).
