# Supplementary material for: Narrow thermal range restricts fertilization and early growth in the habitat‐forming seaweed Durvillaea potatorum (Phaeophyceae)—Implications for aquaculture and climate resilience
Source: J Phycol. 2026 Mar 3;62(2):556–67. doi: 10.1111/jpy.70147 (PMC13103692; doi:10.1111/jpy.70147)
Supplement: Supplementary file 1 — Figure S1. Microscope images of one of the samples from the (a) fertilization experiment, and (b) early germling growth experiment, 24 h after fertilization, indicating ~35–40 and >95% fertilization success, respectively. Fertilization occurred at ~12.5°C in darkness for both samples. [file JPY-62-556-s001.docx]

Figure S1. Microscope images of one of the samples from the (a) fertilization experiment, and (b) early germling growth experiment, 24 hours after fertilization, indicating ~ 35-40 and > 95% fertilization success, respectively. Fertilization occurred at ~ 12.5°C in darkness for both samples.

(a) (b)


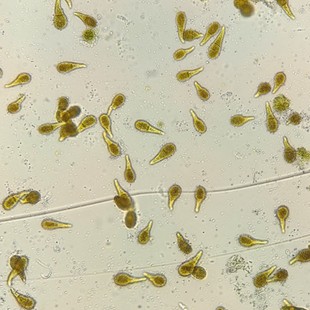

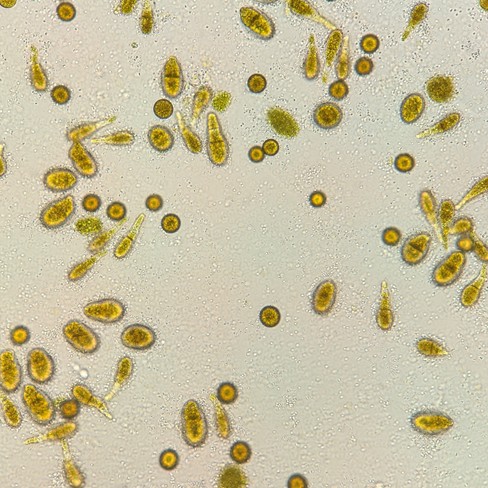


unfertilized eggs

~ 100 µm

fertilized eggs/
germlings
