# Supplementary material for: Narrow thermal range restricts fertilization and early growth in the habitat‐forming seaweed Durvillaea potatorum (Phaeophyceae)—Implications for aquaculture and climate resilience
Source: J Phycol. 2026 Mar 3;62(2):556–67. doi: 10.1111/jpy.70147 (PMC13103692; doi:10.1111/jpy.70147)

Figure S2. All thermal performance curves models fitted to (a) egg release, (b) sperm release, (c) fertilization, and (d) early germling growth of *D. potatorum* at each light level. Nine models were fitted to each response except sperm release, which could not be fitted with a ‘Pawar’ model.

(a)


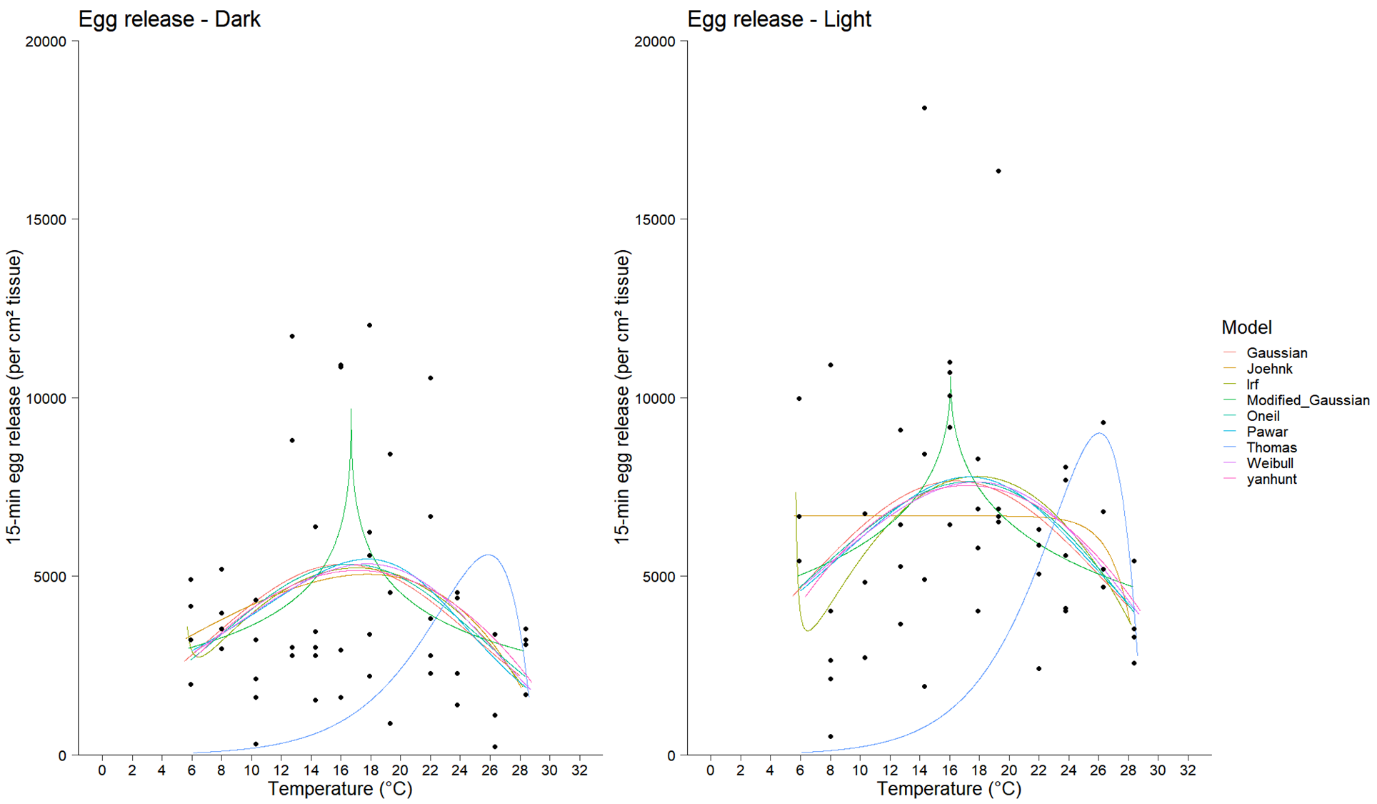


(b)


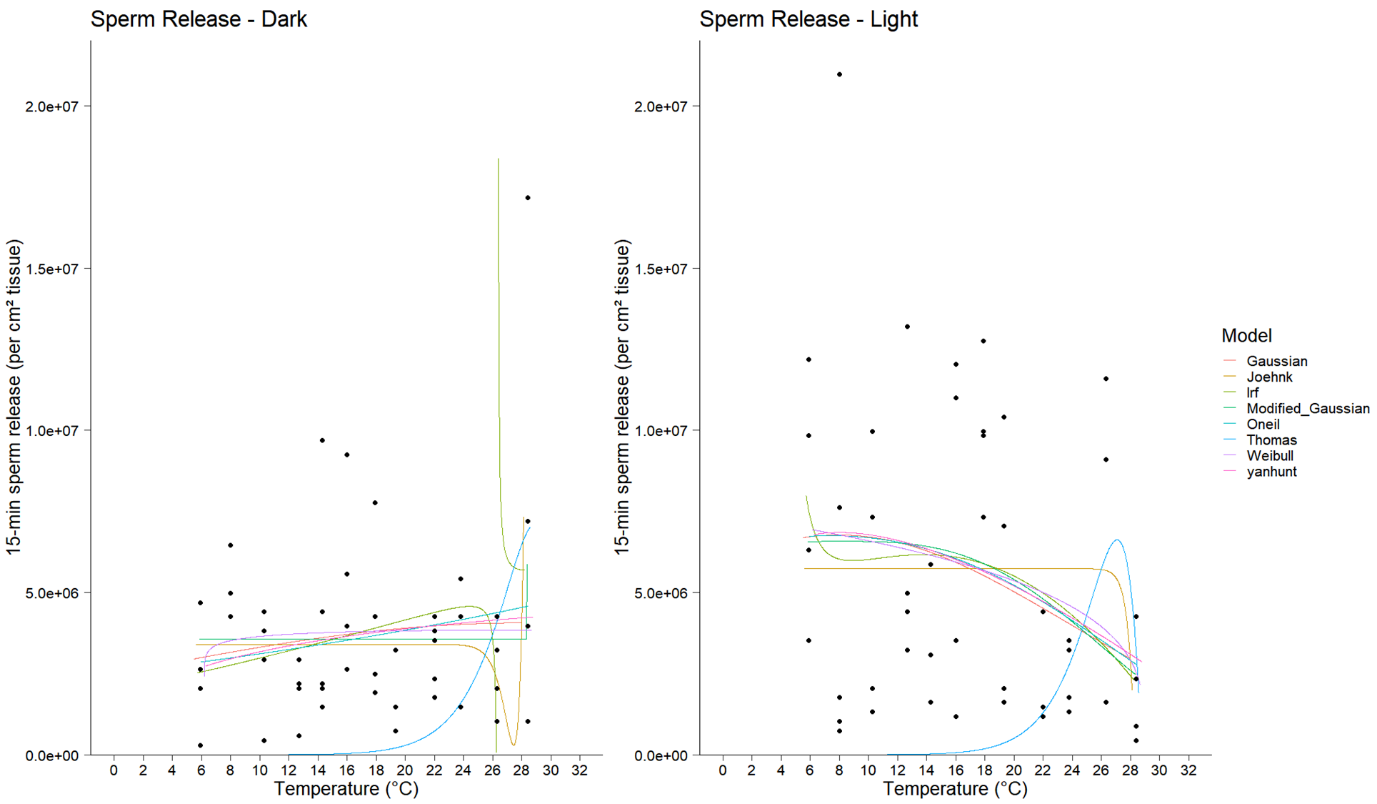


(c)
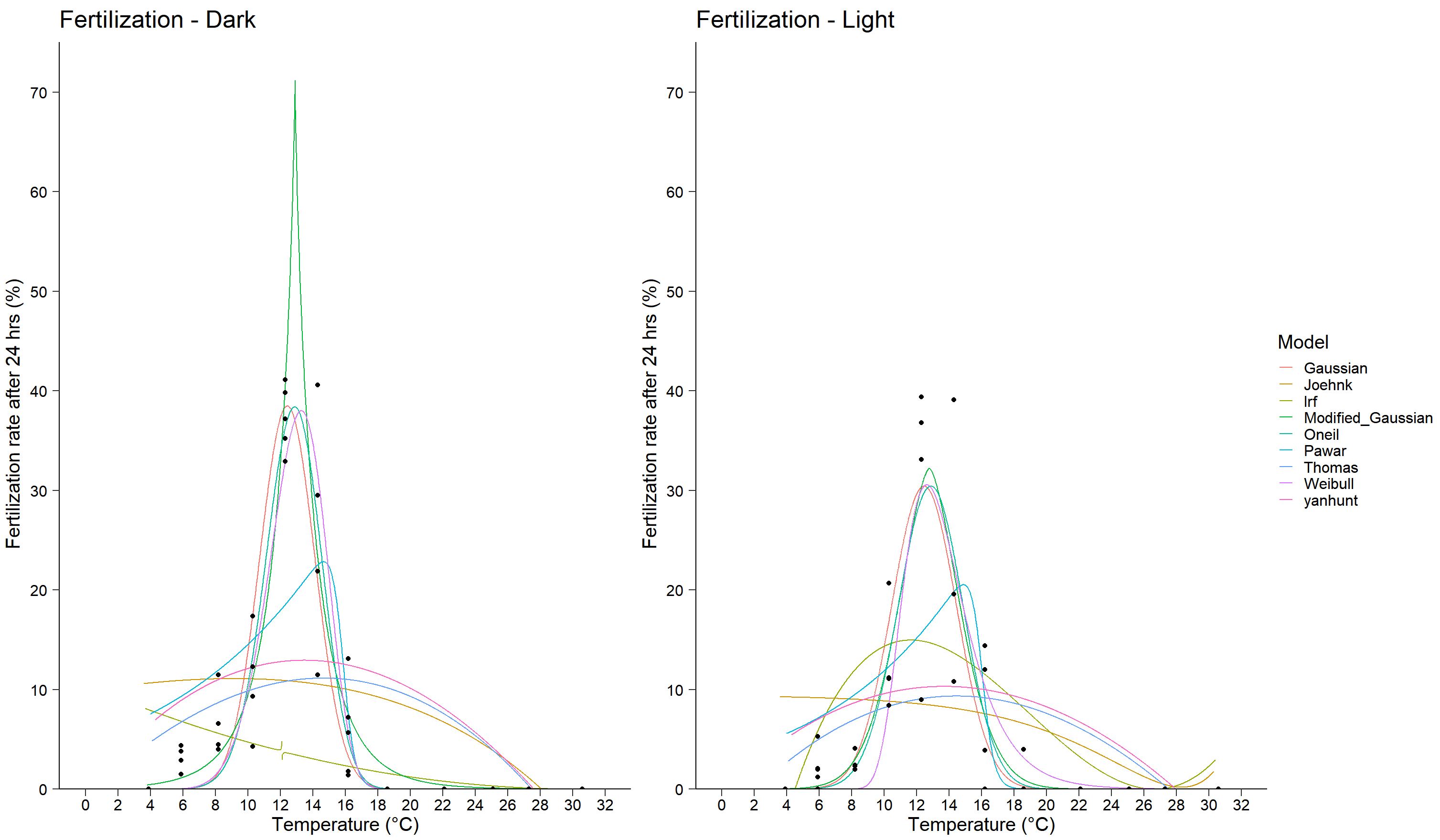


(d)


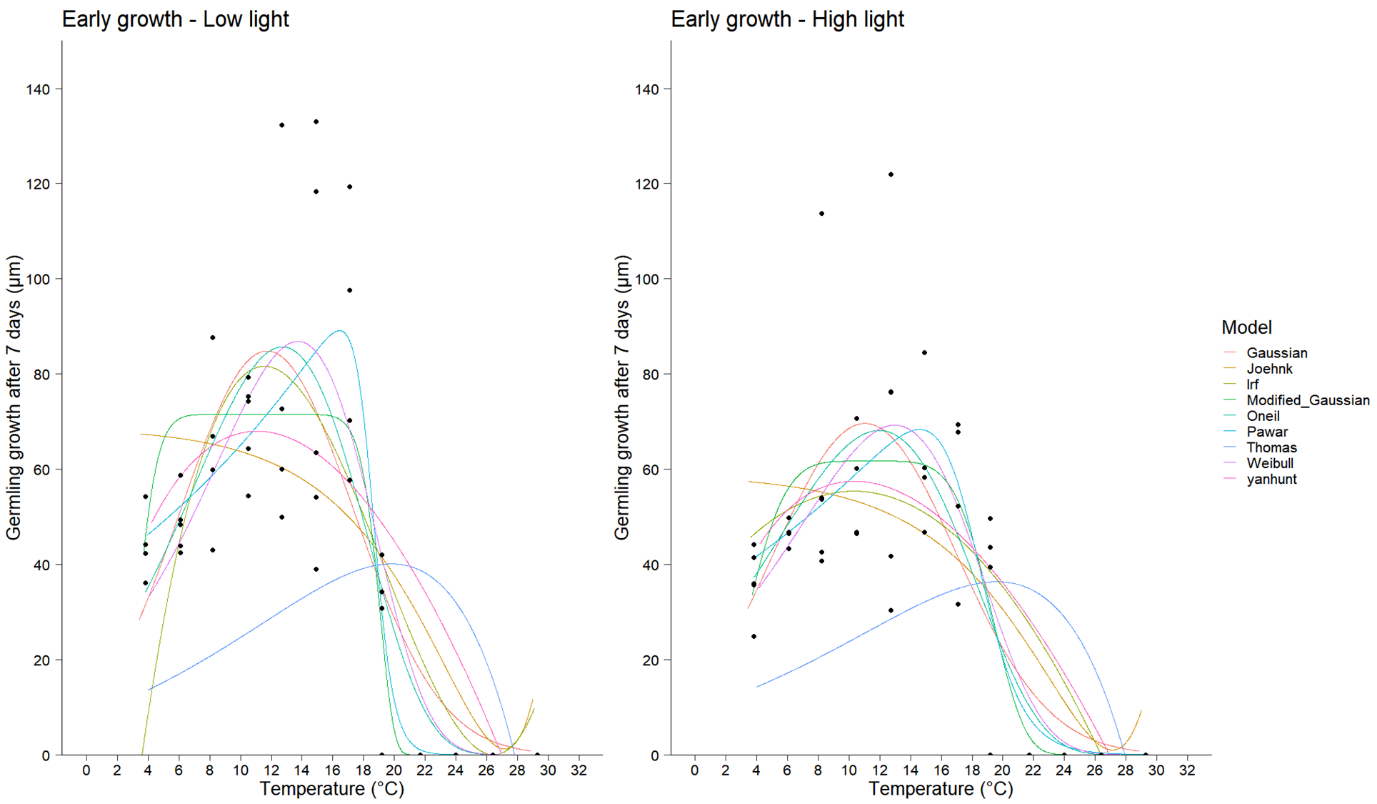

Supplement: Supplementary file 2 — Figure S2. All thermal performance curves models fit to (a) egg release, (b) sperm release, (c) fertilization, and (d) early germling growth of Durvillaea potatorum at each light level. Nine models were fit to each response except sperm release, which could not be fit with a Pawar model. [file JPY-62-556-s002.docx]
