# Supplementary material for: Narrow thermal range restricts fertilization and early growth in the habitat‐forming seaweed Durvillaea potatorum (Phaeophyceae)—Implications for aquaculture and climate resilience
Source: J Phycol. 2026 Mar 3;62(2):556–67. doi: 10.1111/jpy.70147 (PMC13103692; doi:10.1111/jpy.70147)
Supplement: Supplementary file 3 — Table S1. The 12 temperature levels measured in the temperature‐gradient table prior to each experiment. [file JPY-62-556-s003.docx]

Table S1. The 12 temperature levels measured in the temperature gradient table prior to each experiment.

| Experiment | Mean temperature (°C) | | | | | | | | | | | |
| --- | --- | --- | --- | --- | --- | --- | --- | --- | --- | --- | --- | --- |
| Gamete release | 5.90 | 8.03 | 10.33 | 12.73 | 14.27 | 16.00 | 17.90 | 19.33 | 22.00 | 23.80 | 26.33 | 28.43 |
| Fertilization | 3.90 | 5.90 | 8.17 | 10.33 | 12.33 | 14.33 | 16.20 | 18.57 | 22.10 | 25.10 | 27.30 | 30.60 |
| Early growth | 3.77 | 6.10 | 8.23 | 10.53 | 12.73 | 14.87 | 17.10 | 19.23 | 21.67 | 24.00 | 26.43 | 29.30 |
