# Supplementary material for: Narrow thermal range restricts fertilization and early growth in the habitat‐forming seaweed Durvillaea potatorum (Phaeophyceae)—Implications for aquaculture and climate resilience
Source: J Phycol. 2026 Mar 3;62(2):556–67. doi: 10.1111/jpy.70147 (PMC13103692; doi:10.1111/jpy.70147)
Supplement: Supplementary file 4 — Table S2. Details of all thermal performance curve models fitted to egg release, fertilization, and early germling growth of Durvillaea potatorum. The model with the lowest ΔAIC (difference from model with lowest AIC) was preferentially selected. Despite their low ΔAIC values, some models (*) were excluded due to illogical patterns and/or parameter values (see Figure S2) and the model with next lowest Δ AIC was chosen instead. Sperm release was not modeled as none of the fitted thermal performance curve models were adequate or visually representative (see Figure S2). [file JPY-62-556-s004.docx]

Table S2. Details of all thermal performance curve models fitted to egg release, fertilization, and early germling growth of *D. potatorum*. The model with the lowest Δ AIC (difference from model with lowest AIC) was preferentially selected. Despite their low Δ AIC values, some models (*) were excluded due to illogical patterns and/or parameter values (see Figure S2) and the model with next lowest Δ AIC was chosen instead. Sperm release was not modelled as none of the fitted thermal performance curve models were adequate or visually representative (see Figure S2).

| Response | Light level | Model | Δ AIC | *T_opt_* | *R*_max_ | a | b | c |
| --- | --- | --- | --- | --- | --- | --- | --- | --- |
| Egg release | Dark | Gaussian | 0.00 | 16.54 | 5338.27 | 8.93 | NA | NA |
|  |  | yanhunt | 0.54 | 16.73 | 5163.98 | NA | NA | NA |
|  |  | Modified_Gaussian | 0.78 | 16.77 | 10311.66 | 0.79 | 0.35 | NA |
|  |  | Pawar | 1.33 | 17.67 | NA | NA | NA | NA |
|  |  | Weibull | 1.74 | 17.45 | NA | 5347.64 | 107.56 | 12.50 |
|  |  | lrf | 1.89 | 6.65 | 2739.59 | NA | NA | NA |
|  |  | Oneil | 1.96 | 16.62 | 5341.12 | NA | NA | NA |
|  |  | Joehnk | 4.75 | 18.08 | 5055.83 | 4.13 | 1.03 | 1.09 |
|  |  | Thomas | 48.47 | 7.21 | NA | 10.00 | 0.30 | 43.13 |
|  | Light | lrf* | 0.00 | 6.69 | 3475.20 | NA | NA | NA |
|  |  | Modified_Gaussian* | 0.87 | 16.16 | 10701.49 | 4.00 | 0.44 | NA |
|  |  | Gaussian | 1.87 | 16.81 | 7669.12 | 10.47 | NA | NA |
|  |  | yanhunt | 2.47 | 16.79 | 7544.15 | NA | NA | NA |
|  |  | Pawar | 3.43 | 17.22 | NA | NA | NA | NA |
|  |  | Weibull | 3.74 | 17.53 | NA | 7641.29 | 81.79 | 8.07 |
|  |  | Oneil | 3.75 | 17.36 | 7649.14 | NA | NA | NA |
|  |  | Joehnk | 8.94 | 7.14 | 6693.34 | 2.00E-07 | 1.33 | 2.08 |
|  |  | Thomas | 67.43 | 7.06 | NA | 10.00 | 0.32 | 43.44 |
| Fertilization | Dark | Modified_Gaussian* | 0.00 | 13.01 | 71.46 | 0.52 | 0.81 | NA |
|  |  | Gaussian | 3.56 | 12.83 | 38.48 | 1.69 | NA | NA |
|  |  | Oneil | 5.31 | 12.89 | 38.39 | NA | NA | NA |
|  |  | Weibull | 7.39 | 12.99 | NA | 38.00 | 9.46 | 5.75 |
|  |  | Pawar | 72.43 | 14.57 | NA | NA | NA | NA |
|  |  | lrf | 76.62 | 12.13 | 3.94 | NA | NA | NA |
|  |  | yanhunt | 105.87 | 13.08 | 12.94 | NA | NA | NA |
|  |  | Thomas | 109.79 | 13.75 | NA | 10.00 | 0.01 | 27.07 |
|  |  | Joehnk | 115.92 | 9.46 | 11.10 | 4.55 | 1.02 | 1.06 |
|  | Light | Gaussian | 0.00 | 12.86 | 30.43 | 2.00 | NA | NA |
|  |  | Modified_Gaussian | 1.73 | 12.88 | 32.20 | 1.78 | 1.64 | NA |
|  |  | Oneil | 1.99 | 12.88 | 30.43 | NA | NA | NA |
|  |  | Weibull | 5.16 | 12.33 | NA | 30.57 | 186799.82 | -101164.35 |
|  |  | Pawar | 35.67 | 14.78 | NA | NA | NA | NA |
|  |  | lrf | 49.72 | 11.84 | 14.97 | NA | NA | NA |
|  |  | yanhunt | 62.45 | 13.29 | 10.32 | NA | NA | NA |
|  |  | Thomas | 64.04 | 14.63 | NA | 10.00 | 0.00 | 25.43 |
|  |  | Joehnk | 71.42 | 28.62 | 0.20 | 100.00 | 1.35 | 1.23 |
| Early growth | Low | Pawar | 0.00 | 16.33 | NA | NA | NA | NA |
|  |  | Weibull | 9.69 | 13.43 | NA | 86.84 | 521703.83 | 96578.16 |
|  |  | Modified_Gaussian | 11.89 | 11.29 | 71.52 | 7.46 | 10.00 | NA |
|  |  | Oneil | 12.33 | 12.72 | 85.70 | NA | NA | NA |
|  |  | Gaussian | 17.39 | 12.13 | 84.83 | 5.62 | NA | NA |
|  |  | yanhunt | 33.17 | 10.68 | 67.98 | NA | NA | NA |
|  |  | lrf | 33.39 | 11.75 | 81.63 | NA | NA | NA |
|  |  | Joehnk | 41.78 | 27.46 | 1.23 | 100.00 | 1.38 | 1.23 |
|  |  | Thomas | 83.87 | 7.82 | NA | 10.00 | 0.09 | 39.59 |
|  | High | Modified_Gaussian* | 0.00 | 11.45 | 61.69 | 7.35 | 4.99 | NA |
|  |  | Pawar | 0.51 | 14.48 | NA | NA | NA | NA |
|  |  | Weibull | 0.91 | 12.67 | NA | 69.25 | 299786.85 | 49023.42 |
|  |  | Oneil | 1.91 | 11.95 | 68.14 | NA | NA | NA |
|  |  | Gaussian | 4.30 | 11.43 | 69.61 | 5.96 | NA | NA |
|  |  | yanhunt | 18.52 | 9.94 | 57.47 | NA | NA | NA |
|  |  | lrf | 23.58 | 10.51 | 55.37 | NA | NA | NA |
|  |  | Joehnk | 27.11 | 27.43 | 1.00 | 100.00 | 1.36 | 1.21 |
|  |  | Thomas | 70.89 | 4.47 | NA | 10.00 | 0.09 | 46.51 |
